# Supplementary figures and images for: Economic convergence in a globalized world: The role of business cycle synchronization
Source: PLoS One. 2021 Oct 21;16(10):e0256182. doi: 10.1371/journal.pone.0256182 (PMC8530313; doi:10.1371/journal.pone.0256182)

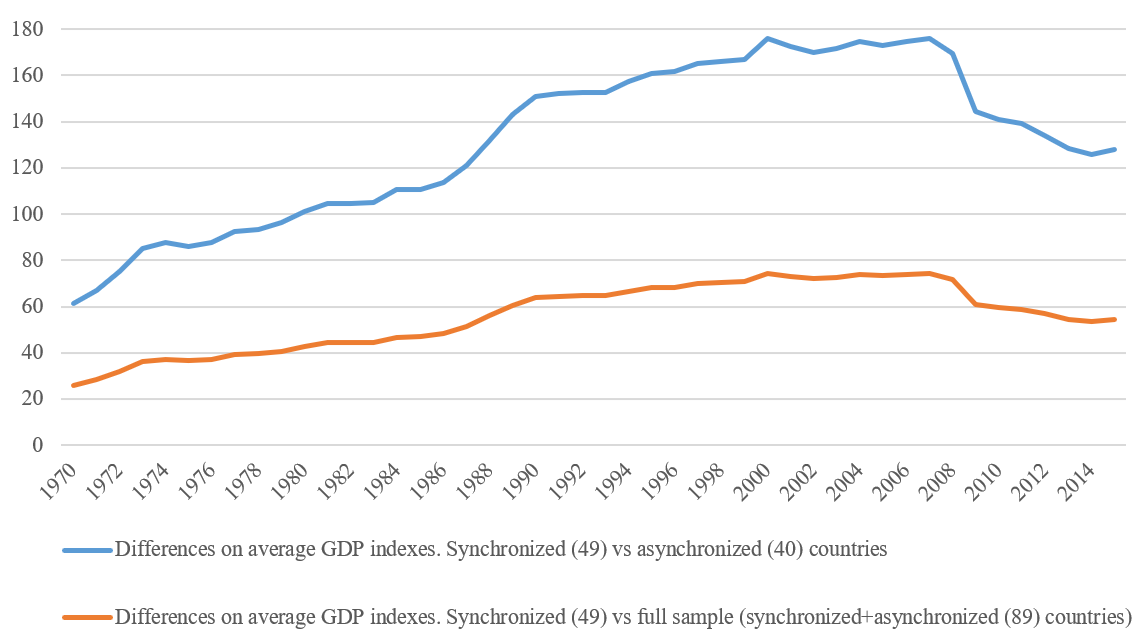

Supplement: S1 Fig — Source: Indexes are own elaboration (Base year is 1950 = 100) from GDP data from the Conference Board Total Economy Database. Synchronized countries are those correlated with the factor explaining global economy fluctuations. (TIF) [file pone.0256182.s001.tif]

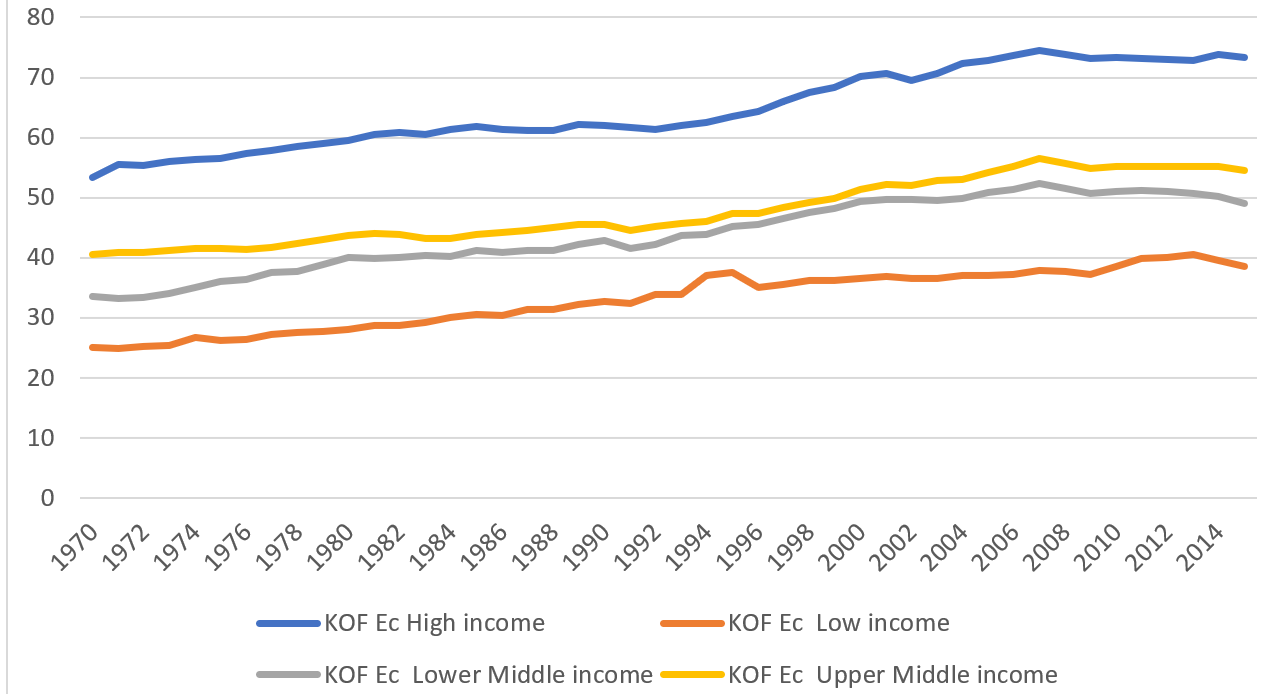

Supplement: S2 Fig — Data source: KOF Swiss Economic Institute. Graph is own elaboration. (TIF) [file pone.0256182.s002.tif]

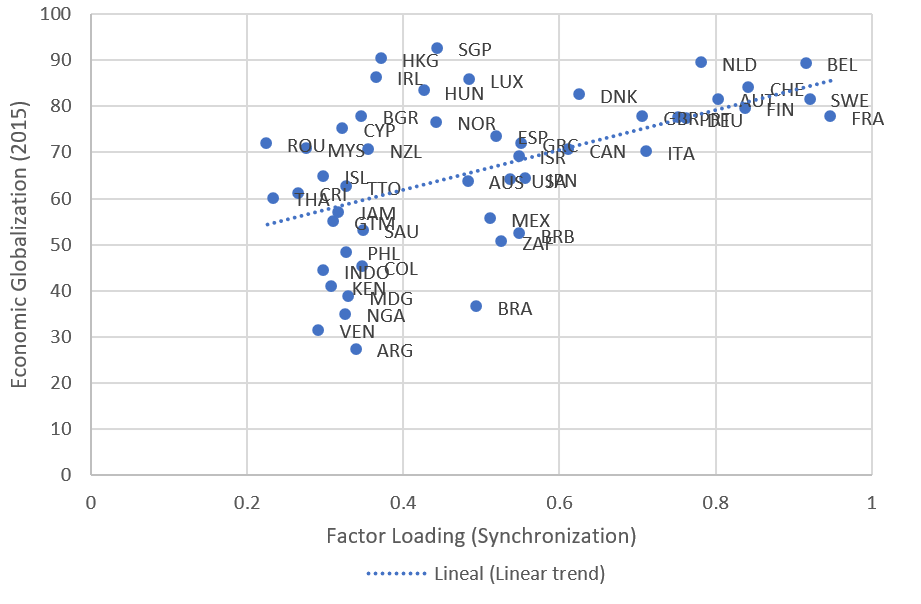

Supplement: S3 Fig — Source: Own elaboration from KOF index and own estimates for synchronization. (TIF) [file pone.0256182.s003.tif]

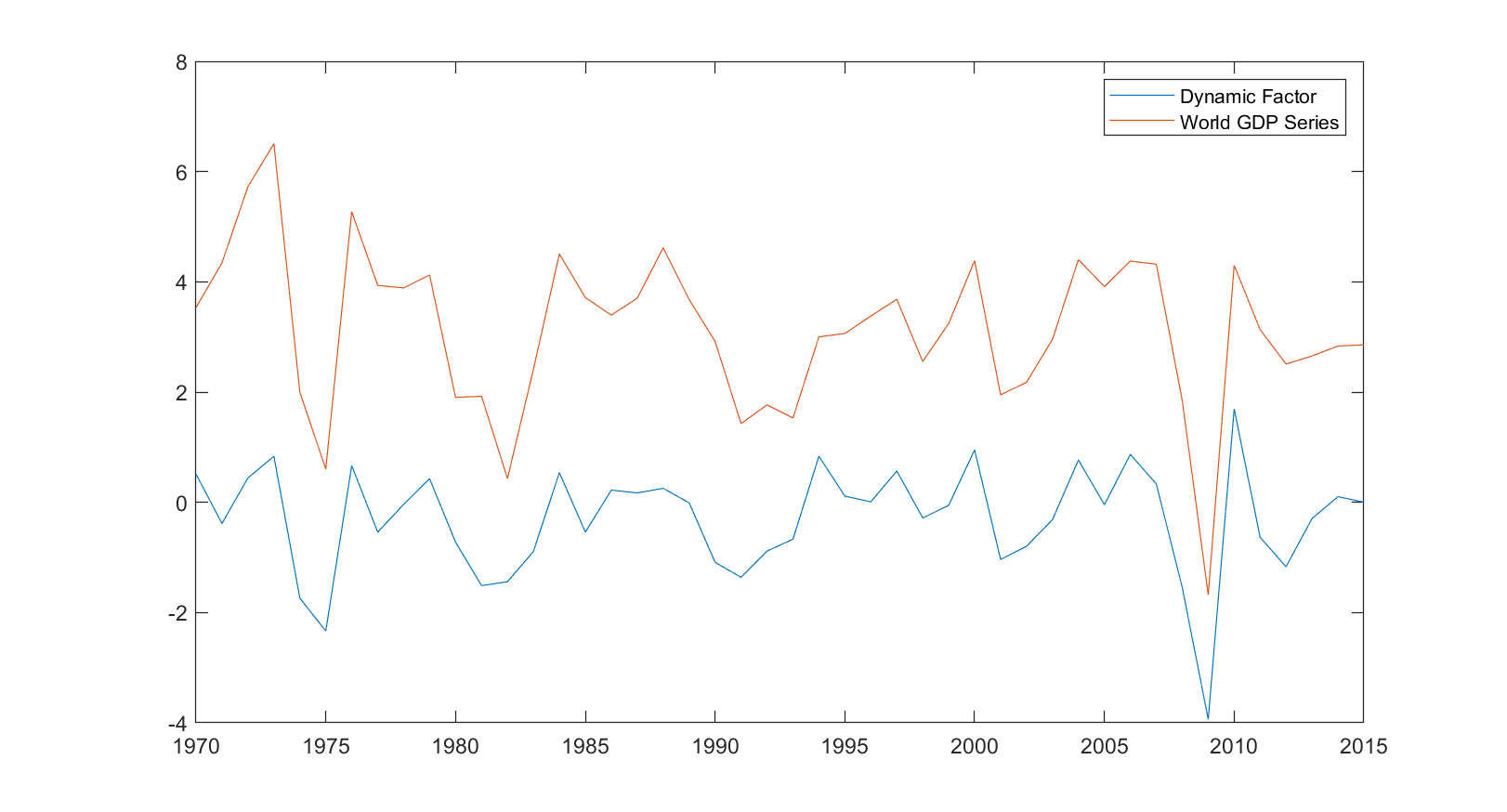

Supplement: S4 Fig — Source: Own elaboration for the dynamic factor and World Bank for World GDP Series. (TIF) [file pone.0256182.s004.tif]
