# Supplementary material for: Economic convergence in a globalized world: The role of business cycle synchronization
Source: PLoS One. 2021 Oct 21;16(10):e0256182. doi: 10.1371/journal.pone.0256182 (PMC8530313; doi:10.1371/journal.pone.0256182)
Supplement: Attachment — Submitted filename: Review Report.pdf [file pone.0256182.s005.pdf]

REVIEW REPORT

Economic Convergence in a Globalized World: The Role of Business Cycle Synchronization

Manuscript Number: PONE-D-21-12597

The manuscript aims to explore the effects of economic globalization on economic convergence for a sample of 89 developed and developing countries in the period 1970-2015 by means of a dynamic panel data model. Overall, the paper approaches a hotly debated topic, being well structured and properly documented. At very first glance, the major concern is depicted by the quantitative approach which is too common. Nevertheless, there occur several shortcomings that should be addressed, as detailed below.

The introductory section presents in a suitable manner the background of the paper, but there is missing the gap in the literature. Which is the novelty and originality of current study? How does present manuscript contribute to the existing literature? Besides, the purpose of the research should be deeply argued.

The section dedicated to earlier literature should be expanded with a table summarizing prior studies. As well, the author(s) should extend the discussion of existing studies on beta-convergence methodology, along with stochastic framework.

With reference to data and methodology, I recommend the author(s) to develop a table regarding the selected variables. As well, I suggest the use of Bayesian panel VAR techniques as baseline approach. Besides, the robustness should be checked through Markov-switching dynamic factor model (MS-DFM) proposed by Diebold and Rudebusch (1996) and Kim and Yoo(1995). In addition, public governance measures should be comprised in the econometric framework.

With respect to the empirical outcomes', the author(s) should compare the results with prior studies. Therewith, the net directional network connectedness should be reported. The sub-section 4.3 is too straightforward and should not be disclosed.

The last section should focus more on policy/practical implications. As well, study limitations should be noticed.

By considering the aforementioned comments and recommendations, I suggest that the paper requires further revisions before being reassessed.
