# Supplementary material for: Economic convergence in a globalized world: The role of business cycle synchronization
Source: PLoS One. 2021 Oct 21;16(10):e0256182. doi: 10.1371/journal.pone.0256182 (PMC8530313; doi:10.1371/journal.pone.0256182)
Supplement: Attachment — Submitted filename: Response to Reviewers.docx [file pone.0256182.s007.docx]

To the editor and reviewers:

To this letter we attach an updated version of the paper. Here we respond to the suggestions from the academic editor and both reviewers. In our answers to reviewers we specify where exactly in the text we have added the changes. We appreciate the comments, since we have improved substantially the content and structure of the paper.

**RESPONSE TO ACADEMIC EDITOR**

1. Please ensure that your manuscript meets PLOS ONE’s style requirements, including those for file naming. The PLOS ONE style templates can be found at

<https://journals.plos.org/plosone/s/file?id=wjVg/PLOSOne_formatting_sample_main_body.pdf> and <https://journals.plos.org/plosone/s/file?id=ba62/PLOSOne_formatting_sample_title_authors_affiliations.pdf>

We changed the format of titles and subtitles, and file naming style (title of the paper).

2. We note that S2 Figure and S3 Figure in your submission contain map images which may be copyrighted. We require you to either (1) present written permission from the copyright holder to publish these figures specifically under the CC BY 4.0 license, or (2) remove the figures from your submission.

We removed maps and plotted economic globalization component from KOF index. Own elaboration graphs, by income level groups (Figure 2). Additionally, we included new Figure 3 (scatter plot) and Figure 4 (factor and World GDP series plotted together) as suggested by the reviewers.

**RESPONSE TO REVIEWERS**

*Reviewer 1:*

1.The introductory section presents in a suitable manner the background of the paper, but there is missing the gap in the literature. Which is the novelty and originality of current study? How does present manuscript contribute to the existing literature? Besides, the purpose of the research should be deeply argued.

We have extended in the introduction section on novelty, contribution to existing literature and research purpose.

2. The section dedicated to earlier literature should be expanded with a table summarizing prior studies. As well, the author(s) should extend the discussion of existing studies on beta-convergence methodology, along with stochastic framework.

Table at the end of literature review. Beta convergence and alternative convergence methodologies section at the end of literature review.

3. With reference to data and methodology, I recommend the author(s) to develop a table regarding the selected variables.

Done. End of section 3.

4. As well, I suggest the use of Bayesian panel VAR techniques as baseline approach. Besides, the robustness should be checked through Markov-switching dynamic factor model (MS-DFM) proposed by Diebold and Rudebusch (1996) and Kim and Yoo(1995).

We consider the Dynamic panel data model the best methodological approach to study income convergence, since it gives us flexibility to add explanatory variables in the context of comparison among group different groups of countries. Additionally, data panel is the methodology used in papers with similar structure to ours (Dreher; Villaverde; Samimi) and we found useful to use a similar methodology, in order to facilitate comparison of results with previous research. The BVAR approach, to our knowledge has not been used in an income convergence setting or to calculate beta convergence, but we find interesting the suggestion and will try to include it in future research as alternative specification to the baseline. We have already found Matlab scripts <https://sites.google.com/site/dimitriskorobilis/matlab/code-for-vars> to develop the BVAR model that could fit in an income convergence model. We have in fact mentioned the BVAR in the limitations in the conclusions section, as a possible extension of the research.

For the suggestion of the factor, we use the GDFM (Generalized Dynamic Factor Model) procedure in a third specification in the robustness checks. M. Forni, M. Hallin, M. Lippi, P. Zaffaroni (2017) “Dynamic Factor Models with infinite-dimensional factor space: Asymptotic analysis”, Journal of Econometrics, 199, 74-92

5. In addition, public governance measures should be comprised in the econometric framework.

We include the political component of the KOF index in the robustness checks. We have extended the definition of this component of the KOF index in second paragraph of section 4.3.

6. With respect to the empirical outcomes’, the author(s) should compare the results with prior studies. Therewith, the net directional network connectedness should be reported.

Last paragraph in section 4.2. compares the results with prior studies.

7. The sub-section 4.3 is too straightforward and should not be disclosed

ok, in appendix.

8. The last section should focus more on policy/practical implications. As well, study limitations should be noticed.

We comment on more policy implications and also limitations in section 5.

*Reviewer 2:*

1.The causal relationship between synchronization, globalization, and growth. The authors hypothesize that business cycle synchronization is a facet of globalization. I have not understood whether this view is expressed as considering the synchronization as a prerequisite via policy channels, or whether it is viewed as a natural and unavoidable process.

We explain in the fifth paragraph of section 5 (conclusions) our view on synchronization as a natural process, for countries that apply economic policy to enhance globalization.

2. An interesting point is that globalization implies higher growth levels but a greater risk of contagion, as well. However, the symmetry of this result is not discussed, i.e., it is not shown whether the gains from globalization in terms of growth are generally greater than the losses during the recessionary phases.

We explain the asymmetry of our results in section 4.2, in the middle of paragraph 3.

3. The discussion of the stylized facts in section 3.1 could be further supported via a scatter plot of the countries according to globalization and synchronization. The clusters that would form on the scatter plot would also be among the countries comprising the global business cycle.

See Figure 3. Thanks for the suggestion.

4. In section 4.1 in the factor loading equation, is there a typo in η_(t-1)?

We have corrected the typo.

6. It would be interesting to include a graph presenting the evolution of the global business cycle in time and compare it with the World GDP growth used in the robustness tests to replicate the results.

See figure 4 for plot of World GDP and the global business cycle estimated.

7. Moreover, it was unclear whether the global business cycle managed to reflect recessionary phases as well.

Yes, it does. See Figure 4 again where co-movement of both series is depicted.

8. In section 5, in the last paragraph it is suggested that the last decade of globalization process, along with the recent COVID recession may alter some of the conclusions of the present work. However, considering the recent recession and its specific character, due to the disruption of global supply chains, wouldn’t we expect a validation of the results? After all, it is argued that globalization implies risks during recessions.

We comment it on the last line of the paper.

9. Would it be possible to include, the list of the countries in the sample in an appendix? While the list of synchronized countries is included in the report of the factor loadings, the extended sample is not outlined.

Thank you for the suggestion, we have included Appendix 1. Lists of synchronized, asynchronized y full sample of countries.

10. The text had a few grammatical errors. I have commented on them on a separate word file, on the text. I am hoping it is not out of place.

We accepted all the grammatical changes proposed and reviewed the text for further improvements in the writing. Thank you.

11. See whether you can update the literature. See for instance:
Holmes, M., Otero, J. and Panagiotidis, T., (2014), A NOTE ON THE EXTENT OF U.S. REGIONAL INCOME CONVERGENCE, Macroeconomic Dynamics, 18, issue 7, p. 1635-1655.

Included in the last paragraph of 2.3, in the extension of the reviewed literature on income convergence.
